# Role of route previewing strategies on climbing fluency and exploratory movements

**DOI:** 10.1371/journal.pone.0176306

**Published:** 2017-04-25

**Authors:** Ludovic Seifert, Romain Cordier, Dominic Orth, Yoan Courtine, James L. Croft

**Affiliations:** 1 Centre d’Etude des Transformations des Activités Physiques et Sportives (CETAPS) - EA 3832, Faculty of Sport Sciences, University of Rouen Normandy, Rouen, France; 2 MOVE Research Institute, Faculty of Behavioral and Movement Sciences, VU University of Amsterdam, Amsterdam, The Netherlands; 3 Centre of Exercise and Sports Science Research, School of Medical and Health Sciences, Edith Cowan University, Perth, Australia; Waseda University, JAPAN

## Abstract

This study examined the role of route previewing strategies on climbing fluency and on exploratory movements of the limbs, in order to understand whether previewing helps people to perceive and to realize affordances. Eight inexperienced and ten experienced climbers previewed a 10 m high route of 5b difficulty on French scale, then climbed it with a top-rope as fluently as possible. Gaze behavior was collected from an eye tracking system during the preview and allowed us to determine the number of times they scanned the route, and which of four route previewing strategies (fragmentary, ascending, zigzagging, and sequence-of-blocks) they used. Five inertial measurement units (IMU) (3D accelerometer, 3D gyroscope, 3D magnetometer) were attached to the hip, both feet, and forearms to analyze the vertical acceleration and direction of each limb and hip during the ascent. We were able to detect movement and immobility phases of each IMU using segmentation and classification processes. Depending on whether the limbs and/or hip were moving, five states of behavior were detected: immobility, postural regulation, hold exploration, hold change, and hold traction. Using cluster analysis we identified four clusters of gaze behavior during route previewing depending on route preview duration, number of scan paths, fixations duration, ascending, zigzagging, and sequence-of-blocks strategies. The number of scan paths was positively correlated with relative duration of exploration and negatively correlated with relative duration of hold changes during the ascent. Additionally, a high relative duration of sequence-of-blocks strategy and zigzagging strategy were associated with a high relative duration of immobility during the ascent. Route previewing might help to pick up functional information about reachable, graspable, and usable holds, in order to chain movements together and to find the route. In other words, route previewing might contribute to perceiving and realizing nested affordances.

## Introduction

Johnson [1] defined skill as the combination of speed, accuracy, form (i.e. economy), and adaptability. Adaptability is a key component for climbing because it provides insights into the on-going co-adaptation of a climber to a set of changing and interacting constraints, which are individually perceived and acted upon. Adapting to different grasping possibilities for a given hold reveals perceptual attunement and calibration of informational variables specifying functional actions. During skill acquisition the learner uses exploratory activities to discover the action possibilities that are afforded by the environment [2]. Gibson suggested, “*perceiving the role of an object* (e.g., holds in climbing) *implies detection of a potential affordance by means of active exploration*. *Using even a simple tool is at a minimum a two-step event–an action that serves as a means to a further step of reaching something desirable*” (p.25) [2]. According to Gibson [3], ‘active exploration’ relates to ‘direct perception’, meaning that the individual does not explore the world by creating and refining a mental representation of the world, but rather perceiving the world is already acting in this world; thus “*to perceive the world is to co-perceive oneself*” (p.141) [3]. When an individual explores, he becomes more and more attuned to information that specifies action. For example, Seifert et al. [4] showed that manipulation of hold orientation during a learning protocol in climbing invited individuals to both exploit their pre-existing behavioral repertoire (i.e., horizontal hold grasping pattern and trunk face to the wall) and to explore new behaviors (i.e., vertical hold grasping and trunk side to the wall). Assuming that adaptability can be assessed through the ability to perceive and realize affordances (opportunities for action) in a performance environment [3], analysis of the relation between *exploratory* and *performatory* movements could explain how climbers perceive ‘climbability’ of a surface and exploit environmental properties to act [5–7]. Gibson [3] suggested that “*slopes between vertical and horizontal afford walking*, *if easy*, *but only climbing*, *if steep and in the latter case the surface cannot be flat; there must be holds for the hands and feet*” (p.132). Therefore, a climbing wall could be a barrier to pedestrian (i.e. bipedal) locomotion but this wall could be ‘climbable’ when quadrupedal locomotion is used. Thus, climbability refers to the perception of an approximately vertical surface and its layouts (e.g., holds for hand and feet) leading humans to reach, grasp, stand, and use holds as a support for quadrupedal locomotion. During indoor wall climbing, Pijpers et al. [7] distinguished exploratory and performatory movements of potential holds on a rock surface by holds that were used or not used as support during the ascent. Calculating the ratio between ‘touched/grasped’ and ‘used’ holds to move upward could indicate how climbers explore environmental properties by using adaptively perception and action systems.

The distinction between touched, grasped, and used holds is also used in lead climbing competition to assess achievement when a competitor falls before reaching the top. The International Federation of Sport Climbing (IFSC) rules indicate that “*a hold shall be considered as “controlled” where a competitor has made use of the hold to achieve a stable or controlled position*, *whereas a hold from which a competitor has made a controlled climbing movement in the interest of progressing along the route shall be considered as “used”*. To help the judges make their decision, the IFSC rules indicate that “*a controlled climbing movement may be either “static” or “dynamic” in nature and in general will be evidenced by (i) a significant positive change in position of the competitor's center of mass; and (ii) the movement of at least one hand in order to reach either the next hold along the line of the route*”. These rules reinforce the idea that repetitive exploratory movements may lead to early fatigue, stoppages, and falls. This also places into perspective why skilled climbers try to minimize their exploratory actions at the limbs during climbing, for example the ‘three-holds-rule’, a commonly used training constraint [8], limits touching to fewer than three surface holds before grasping a functional one. Previous studies have shown how *route* and *hold designs* induce more or less exploratory behaviors [4] and functional performatory movements [9].

Information that specifies action is sometimes hidden and climbers can pick up misinformation information during the climb. “*If misinformation is picked up*, *misperception results*” (p.142) [3]. Once a climber has started a route it can be difficult to climb down or to change his/her path. Visual inspection of the route before the climb (route previewing) may enhance climbing performance by giving the climber the opportunity to perceive affordances offered by the surface and its layout, and thus to minimize misperception [10]. During route previewing, climbers might simulate how to grasp each hold and sequences of holds, to find the route. Whilst simulating climbers move along the climbing wall to look at the hold shape from different points of view. In lead climbing competition, climbers have time for route preview but they climb the route in ‘on-sight’ condition, i.e. without prior physical practice before attempting to ascend. Route previews are typically undertaken at the bottom of an ascent and IFSC rules mention, “*Competitors may use binoculars to observe the route*, *and make hand-drawn sketches and notes*” (p. 25). Even with binoculars the quality of optical information of the holds is not uniform and invariant features may be more difficult to pick up at the higher parts of the climb. For instance the line of sight makes it more difficult or impossible to view overhand edges.

Boschker et al. [5] and Pezzulo et al. [11] provided support for the role of route preview in identifying affordances for climbing. In both studies, climbers were required to preview certain features of the climbing route (such as position and orientation of holds) and reproduce them. Expert climbers typically focus on functional features the wall supported (such as hold grasping and sequences of movements). Since more than one hold is generally required to perform climbing actions this presumably facilitated the recall of more ‘chunks’ of information (in this case multiple holds associated with action(s)). Whereas, the inexperienced climbers did not recall such clustered information, mostly reporting the structural features of the holds (such as their orientation and shape) [10]. In these respects there is good evidence that the perception of opportunities for action during route previewing is associated with more effective recall [5,11]. However, neither preview nor subsequent climbing behaviors were examined directly in these studies. Sanchez et al. [12] examined the influence of a route preview (with and without preview) on the output (route completion) and form (number and duration of moves and stops) of a subsequent climb. Results indicated that route preview did not influence output performance but did influence the form—climbers made fewer, and shorter stops during their ascent following a preview of the route [12]. The influence of preview was more obvious in expert climbers who paused less frequently and took shorter periods at rest regions [12]. These findings indicate that route previewing might contribute to climbing fluency (i.e. climbing by minimizing the number and duration of saccades and stops [13]). Fluency could be captured by spatial (e.g. geometric index of entropy [14]), temporal (e.g., ratio between immobility and motion [15]) and spatial-temporal measurements (e.g., jerk coefficient corresponding to the smoothness of the hip displacement [13])—for a review, see [16].

More direct measures of visual perception have provided further insight into the visual-motor skills used during preview. A recent study measuring gaze behavior during route preview highlighted four visual strategies used by skilled climbers [17]: (i) *Ascending strategy*: where the climber looks from the bottom upwards and finishes the preview on the top hold; (ii) *Fragmentary strategy*: where the climber looks at parts of a route and ignores a lot of holds and quickdraws; (iii) *Zigzagging strategy*: that involves moving gaze from side to side (hand holds to foot holds) as the climber looks through the route; and (iv) *Sequence-of-blocks*: where the climber gradually looks through the route in blocks of two to four hand holds, with particular attention being focused on crux points (i.e. the most difficult parts of the route). This sequence-of-blocks strategy was used by 52% of the climbers when they previewed a route of an intermediate difficulty and by 87% of the climbers for a route with an advanced level of difficulty [17]. Grushko and Leonov [17] suggested that sequence-of-blocks relate to more in-depth visual analysis whereas ascending strategy is mostly connected with getting to know the route overall. Button et al. [18] suggested that ‘simple’ previews may initially be used to find the location of potential handholds (and footholds), whereas more ‘sophisticated’ previews are necessary to chain movements. Button et al. [18] noted that the study of Grushko and Leonov [17] is the only published data directly examining eye movements during preview; however, it failed to provide a theoretical and methodological rationale to compute which visual strategies were used. Based on the ecological approach to visual perception [3], it could be hypothesized that sequence-of-blocks strategy relates to in-depth visual inspection of how to reach, grasp, or use the hold and might reveal how an individual perceives climbing affordances. Similarly, a zigzagging strategy might relate to how an individual perceives the transition between holds, and the coordination between using handholds and footholds. Finally, ascending and fragmentary strategies are used to find the overall route when reaching and grasping holds are not an issue. Thus, sequence-of-blocks and zigzagging strategies would be associated with deep visual inspection, probably because the informational variables (e.g. hold shape, orientation, size, etc) of the route don’t clearly specify action. Conversely, ascending and fragmentary strategies would be associated with superficial visual inspection, probably because the climber can perceive the functional features of the holds and the sequence of movements that are required. These hypotheses about perceiving and realizing affordances in climbing remain untested. Consequently, the aim of this study was to understand the gaze behavior during pre-visual inspection of a climbing route, with a special focus on route previewing strategies; in particular this study aimed to understand whether climbers using certain route previewing strategies were more likely to perceive and to realize climbing affordances. We used cluster analysis to investigate the various profiles of gaze behavior during route previewing and their relationships with climbing fluency and exploratory movements during the climb. We hypothesized that sequence-of-blocks and zigzagging strategies (reflecting deep visual inspection) during route previewing might be associated to higher exploratory movements and lower fluency during the climb.

## Materials and methods

### Participants

Ten experienced climbers (mean age of 27.6±7.2 y; mean height: 175.4±5.1 cm; mean weight: 62.5±6.8 kg; mean climbing experience of 11.2±6.6 y) and eight inexperienced climbers (mean age of 20.3±2.2 y; mean height: 167.9±9.0cm; mean weight: 64.1±10.9 kg; mean climbing experience of 3.0±0.0 y) volunteered to participate in this study. Climbers were classified as experienced if they climbed at a grade between 6c and 7b+ on the French Rating Scale of Difficulty (F-RSD) (the scale ranges from 1 to 9) [19]. The inexperienced climbers had practiced indoor climbing for three hours per week for two years with a self-reported climbing grade of 5 or 5+ on the F-RSD, which corresponds to an intermediate level of climbing ability [20]. Participants had a healthy body mass index range (<25) and an arm span of at least 140 cm.

### Protocol

Participants were allowed up to three minutes to preview a climbing route. If they chose to take less than three minutes they indicated this to the experimenter. They were told to self-pace their ascent and climb as *fluently* possible, i.e., without falling down and by minimizing pauses and rest periods of body displacement during the ascent [4,13,14]; noting that speed (i.e., climbing fast) is not a criteria of fluency. We did not give specific instructions so that perception and action processes could emerge during exploratory behaviour during the preview and follow-up climb. The route was top-roped, i.e. the route was climbed with a rope anchored above the climber at all times to avoid stoppages that are required during lead climbing where the climber has to clip into quickdraws.

**Fig 1:**
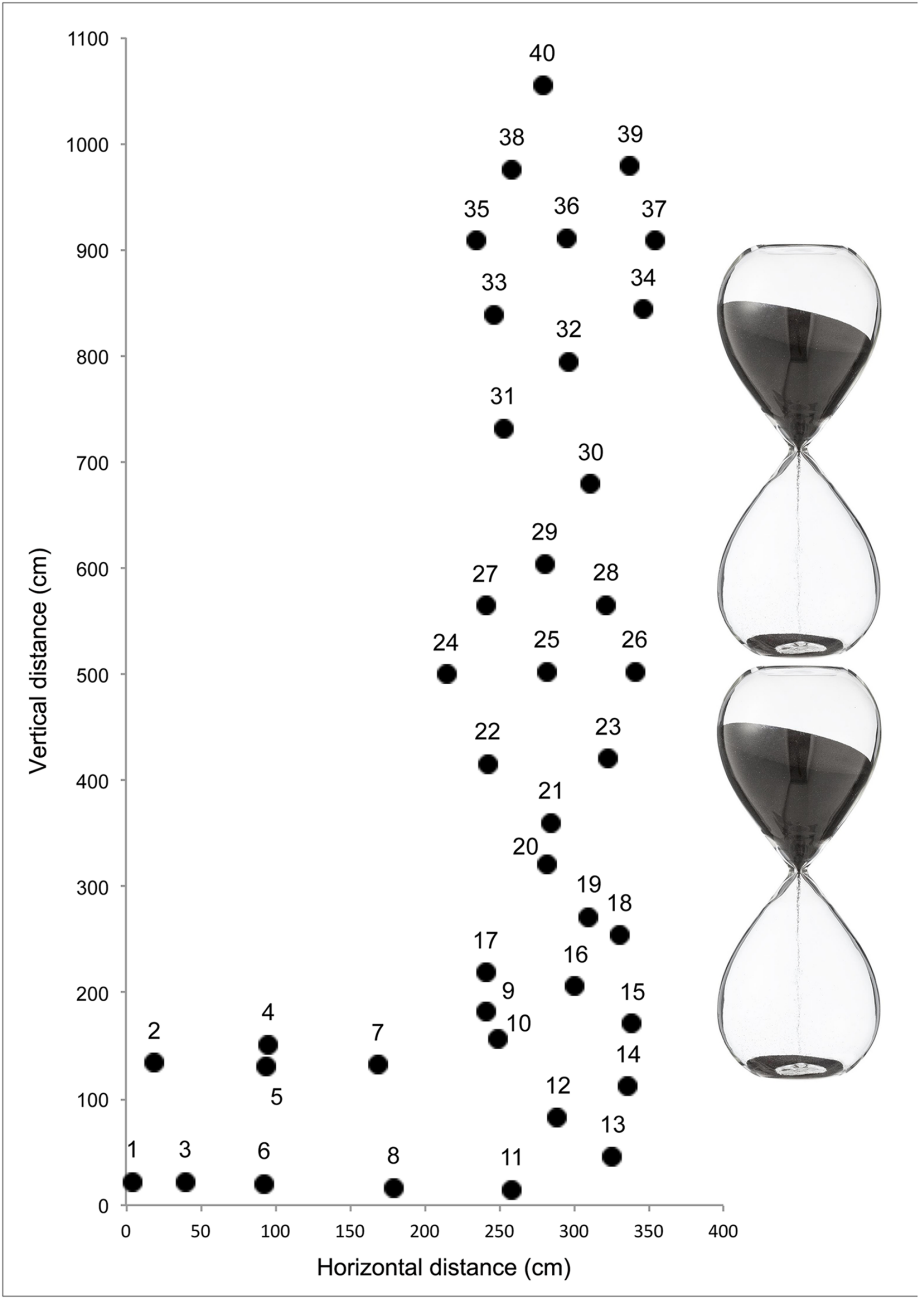
Map of the climbing route with the X and Y coordinates of the 40 holds.

The route consisted of a 4.0 m horizontal traverse followed by a 10.3 m vertical ascent on an artificial indoor climbing wall composed of 40 holds, including 8 footholds and 32 handholds, which were bolted to a flat vertical surface (Fig 1). The distance between holds was never more than 1 m horizontally and 1 m vertically to ensure that the arm-span of all participants enabled them to reach the holds as intended. The holds were identifiable by a green color and their locations were identified prior to previewing with a laser pointer. Participants were instructed not to preview during this demonstration. The holds were placed by three professionally-certified route setters who ensured that the route matched a low grade of difficulty; rated at 5b on the F-RSD. The grade of the route was not provided to the participants and they were asked to estimate the grade at the end of previewing. The holds were set in a pattern similar to two hourglasses to allow various climbing paths to the top (Fig 1).

### Ethics statement

This study was performed in accordance with the Declaration of Helsinki and was approved by the Institutional Review Board and Ethics Committee of the Faculty of Sport Sciences, University of Rouen Normandy. Procedures were explained by a written document to the participants, who then gave their written informed consent to participate.

### Data collection

#### Gaze behavior during preview

Eye tracking glasses (SIMI, version 2.0, SensoMotoric Instruments Inc., Teltow, Germany) recorded binocular eye gaze behavior via a wireless connection to a Samsung Galaxy Note 4 (placed in the climber’s chalk bag) in real time at a sampling rate of 60 Hz, with gaze tracking range of 80° horizontal and 60° vertical, and gaze tracking accuracy of 0.5° over all distances. The scene camera located between the eyes records at 960 x 720 pixels with field of view: 60° horizontal, 46° vertical, and a sampling rate of 30 Hz (www.eyetracking-glasses.com). A three-point calibration was used before each trial, and at the end of each trial the climber fixated the last hold so we could check for any offset due to movement of the glasses during the climb.

#### Climbing fluency, exploratory and performatory movements of limbs

Inertial measurement units (IMU) were attached to the right and left wrist, right and left foot, and hip as previously done by Seifert et al. [21]. An IMU was also placed on the climbing wall to locate it in the Earth reference frame. These IMUs include a combination of a tri-axial accelerometer (±8G), tri-axial gyroscope (1600°.s^-1^) and a tri-axial magnetometer (*MotionPod*, Movea, Grenoble, France) and sample at 100 Hz. Wi-fi transmissions to a controller allow recording using *MotionDevTool* software (Movea, Grenoble, France). These IMUs have been used previously to assess climbing fluency using the jerk coefficient of hip trajectory [13] and for automatic detection of activity states during climbing [22]. Synchronization between the eye tracking system and IMUs was done by three handclaps during which the climber watched his hands; the hand clapping was detected in the scene camera of the glasses and through the acceleration peak of the IMU located on the wrists.

### Data analysis

#### Gaze behavior

Gaze behavior during the preview was analyzed using SMI BeGaze eye tracking analysis software (www.smivision.com). Visual fixations were automatically detected from spatial and temporal characteristics of gaze data, under the assumption that fixation points generally occur near one another [23]. The maximum spatial dispersion was set at 100 pixels. Since fixations are rarely less than 100 ms, and often in the range of 200–400 ms [23], we set the minimum fixation duration at 90 ms. The dispersion-based algorithm identifies fixations as groups of consecutive points within a particular dispersion, or maximum separation. It uses a moving window that spans consecutive data points checking for potential fixations. The moving window begins at the start of the trial and initially spans a minimum number of points, determined by the given minimum fixation duration (90 ms) which is equivalent to three frames for the sampling frequency (30 Hz). The algorithm then checked the dispersion of the points in the window by summing the differences between the points' maximum and minimum *x* and *y* values; in other words, dispersion D = [max(*x*)—min(*x*)] + [max(*y*)—min(*y*)] (SMI BeGaze, p320-321). If the dispersion was above 100 pixels, the window did not represent a fixation, and the window moved one point forward. If the dispersion was below the maximum dispersion value, the window represented a fixation. In this case, the window was expanded forward until the window's dispersion exceeded the spatial threshold. The fixation was registered at the centroid of the final window with the given onset time and duration.

Previous research has shown that inexperienced performers exhibit fixations of shorter duration but a higher number of fixation than experts [18,24,25]. As the duration of route preview influences the number and total duration of fixations, we calculated the *relative duration of visual fixations as a percentage of the route preview duration*, and the *search rate (ratio between the total number of fixations and the total duration of the fixations*; for more details, see [26]). The 40 holds of the route were defined as 40 areas of interest (AOI). Next we calculated the *percentage of visual fixations on each AOI vs*. the *percentage of visual fixations on the climbing wall*, and the *average duration of visual fixations on holds* (in seconds) *vs*. *the average duration of visual fixations on the climbing wall* (in seconds).

**Fig 2:**
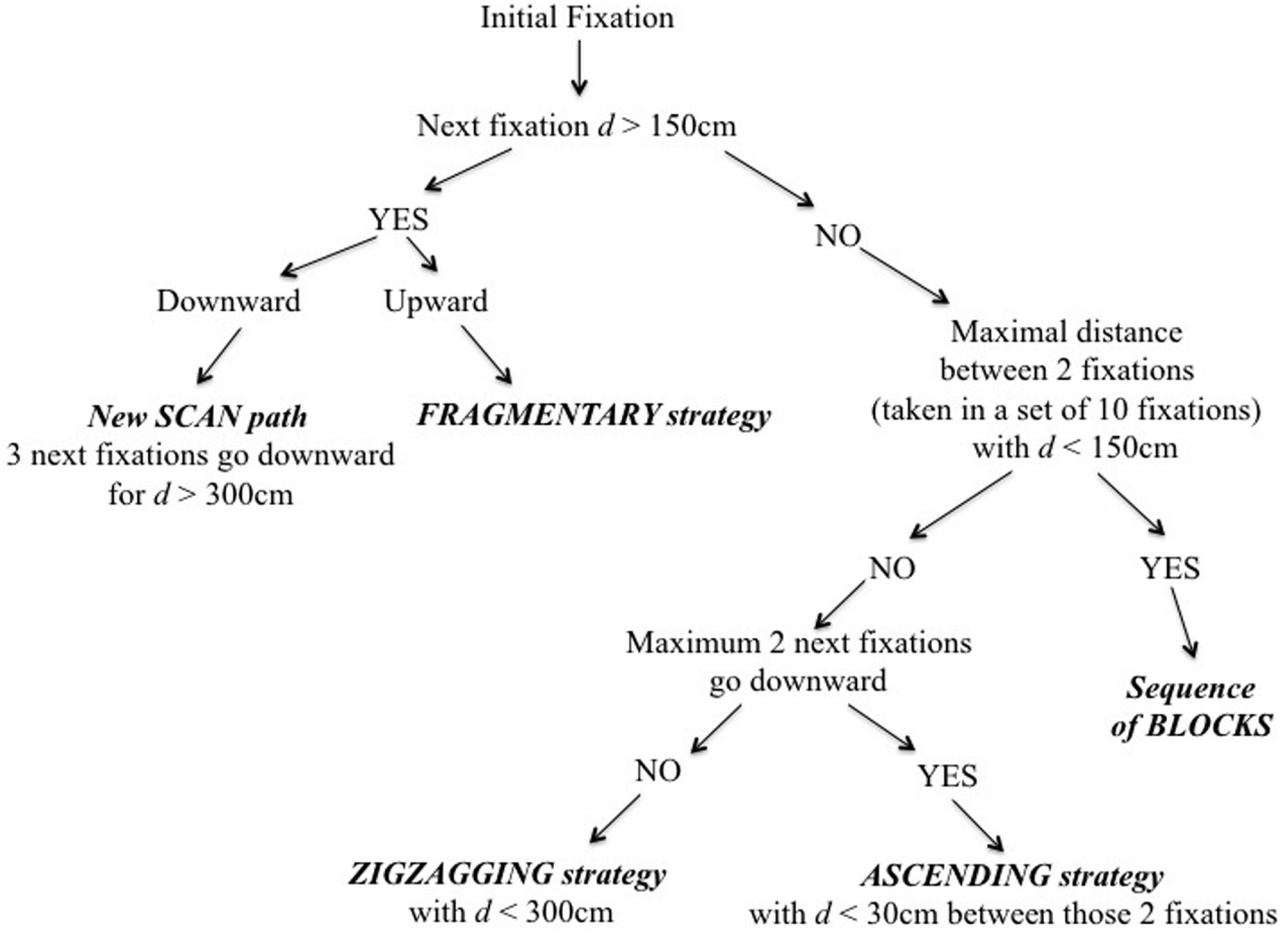
Decision tree to define the visual strategies during the route preview.

A previous study observed several visual strategies during preview of climbing routes: *fragmentary strategy*, *ascending strategy*, z*igzagging strategy* and *sequence-of-blocks* [17]. One aim of this study was to detect these visual strategies using an algorithm that could automatically recognize each visual strategy using a decision tree (Fig 2).

From an initial fixation, the aim was to classify the subsequent fixation sequence depending on the fixation locations. The first step of the decision tree concerns the distance to the next fixation. If the next fixation is greater than 150 cm away and in an upward direction it is classified as a *fragmentary strategy*. If the total distance of the next three fixations is greater than 300 cm away and in a downward direction this is considered a new *scan path*. When the gaze partially or totally comes back to a previous section of the route or back to the start of the route, this can occur in up to three fixations. We considered that these fixations should not be less than 3 m, which is greater than the arm span.

An ascending strategy corresponds to “*look from below to upward and finish preview on the top hold*” ([17], p.172). This strategy gives a visual overview with enough fixations (we set the threshold at ten fixations) without too many downward scans (threshold number set at two fixations). During an ascending strategy it is reasonable to assume that downward fixations cannot be greater than the arm size; so we set the threshold distance for downward visual fixation at 30 cm vertically.

When the maximal distance between two fixations (taken in a set of ten fixations) is greater than 150 cm and contains more than two fixations going downward (without exceeding a distance of 300 cm because that defines as a new scan path), this defines a *zigzagging strategy*. The rationale for setting the maximal distance of 150 cm was that a zigzagging strategy might be used to search for handholds and footholds that typically fall within this distance.

When the maximal distance between two fixations (taken in a set of ten fixations) was below 150 cm, it was defined as a *sequence-of-blocks*. The threshold distance was set at 150 cm because it corresponds to the maximal distance between two holds that a climber could reach within a quadrilateral composed of four handholds. When a climber visually fixates an area of up to four hand holds for ten fixations, this area of the climb is defined as a ‘crux’ area, i.e. the most difficult section [17].

The last step was to test this classification system on three participants and compare the outcome to classifications derived from manual annotation by three researchers. The three participants were selected because their visual strategies could be easily isolated from each other and recognized by the researchers. The threshold distances within the decision tree were adjusted until the automatically obtained strategies matched the known strategies, in the same way that activity states in climbing have been defined previously [22].

#### Climbing fluency

Climbing fluency was assessed by jerk coefficient which indicates the smoothness of hip trajectory [13]. The jerk coefficient was calculated from the 3D orientation of the hips in the Earth reference frame (as described in [13]).

#### Exploratory and performatory movements of limbs

The IMU sensors were used to detect the different activities of the climber as reported previously [22]. Based on the four limbs and the hip, and depending on whether the limbs and/or hip were moving or immobile, four climbing states were identified: immobility (i.e., all limbs and the hip are immobile), postural regulation (i.e., all limbs are immobile and the hip is moving), hold interaction (i.e., at least one limb is moving and the hip is immobile), traction (i.e., at least one limb is moving and the hip is moving). The immobility state represents the state when the climber is not moving; he might be resting due to fatigue or might be looking at the route to determine the climbing path. The postural regulation state represents an adjustment of the climber’s center of mass while the limbs stay on the same holds. It might consist of a body rotation so he/she could reach a hold that would not be reachable from his/her previous body configuration [22]. In the traction state the climber is moving (generally upward) using at least one limb, previously characterized as *performatory* movements [7]. The hold interaction state represents movement of a limb while the hip (and therefore the global position of the climber on the wall) remains immobile. It could correspond to at least three behavioral states [22]: a change of the way a hold is used before the next traction, i.e., a transitional hold movement; a change of the position and orientation of the hand/foot on an hold so it can be used differently; repetitive movements with a limb to determine which hold is the most appropriate to use for the next traction. For clarity, this fourth activity state was split into two sub-states named *hold change* vs. *exploration* (i.e., *exploratory* movements [7]).

The ratio between exploratory and performatory movements could explain how climbers pick up affordances–opportunities or invitations for action [3,27], i.e. how climbers perceive climb-ability of the environment and exploit environmental properties to allow action [5–7]. Nieuwenhuys et al. [26] showed that exploration occurs not only at the motor level, but also at the visual level for route finding or hold reaching and grasping when the climber is immobile or is regulating his posture. Therefore, the time spent immobile or during postural regulation could potentiality reflect a part of exploratory activity. In summary, the capability of picking up affordances for climbing behaviors could be approached by examining the relationships between gaze behavior during route previewing (e.g., duration of preview, number and duration of fixations, types of visual strategy) and behavioral feature during the climb (e.g., climbing fluency such as jerk coefficient, relative duration of immobility, exploratory *vs*. performatory movements).

### Statistical analysis

We attempted to identify different profiles of route previewing using cluster analysis. Then, we used Pearson correlation tests to identify associations between route previewing variables (found meaningful by the cluster analysis) and climbing variables (i.e., climbing duration, climbing fluency and relative duration of the different activity states in climbing).

#### Cluster analysis

Profiles of gaze behavior during route preview were defined through hierarchical cluster analysis (HCA) with the squared Euclidean distance dissimilarity measure and the Ward linkage method [28–30]. Thirteen variables were used to classify the climbers. As explained earlier, the participants had up to three minutes to preview the climbing route. Comparing the number and the duration of visual fixations would be affected by the preview duration, so instead we calculated the *ratio between the number of fixations and the duration of the preview*, the *relative duration of visual fixations as a percentage of route preview duration*, and the *search rate (ratio between the total number of fixations and the total duration of the fixations)*. Then, as the climber can fixate on the holds or the climbing wall, we included four other measures: the *percentage of visual fixations on holds*, the *percentage of visual fixations on the climbing wall*, the *average duration of visual fixations on holds* (in seconds) and *the average duration of visual fixations on the climbing wall* (in seconds). The last five measures refer to the visual strategies during the preview: the *number of scans*, the relative duration of the *fragmentary strategy*, *ascending strategy*, z*igzagging strategy* and *sequence-of-blocks* (expressed in percentage of the route preview duration). Each variable was standardized (i.e., values are scaled within 0 and 1) to avoid overweighting due to different scales. The results of the cluster analysis were described as a dendrogram.

#### Cluster validation

The Calinski-Harabasz (CH) index was used to estimate the number of clusters that best fitted the data and thus best classified the participants in each cluster [31]. This index takes a form of Index = (a*Separation) / (b*Compactness), where *a* and *b* are weights [32,33]. In particular, CH (*k*) = (B(*k*) / (*k*—1)) / (W(*k*) / (*n*–*k*)) where *n* represents the number of participants, *k* denotes the number of clusters, and B(*k*) and W(*k*) denote the between and within clusters sums of squares of the partition. The maximal value of this index indicates the optimal ratio between inter-cluster distance (i.e., inter-cluster separation) and intra-cluster distance (i.e., intra-cluster compactness) and in turn, an optimal number of clusters [28,30,33]. The CH index was calculated from two to ten potential clusters.

Finally, classification of the variables which differentiated the clusters was determined by a bootstrapping procedure [34]. The bootstrapping procedure to construct the dendrogram, was repeated 12 times (number of variables– 1) excluding one variable at the time, to examine whether the classifications were stable. For example, if variable 1 was removed and the number of cluster remained the same and if each participant remained in his initial cluster then the variable did not contribute to the discrimination; when exclusion of the variable led to a modification of the number of clusters and/or the classification of the participant in the cluster (i.e., number of participant switching) that variable was considered important in discrimination. All tests were conducted with Cluster Validity Analysis Platform (CVAP) (Version 3.7) [35] with Matlab (R2014a, 1994–2014, MathWorks Inc, Massachusets).

## Results

### Gaze behavior during route preview

**Fig 3:**
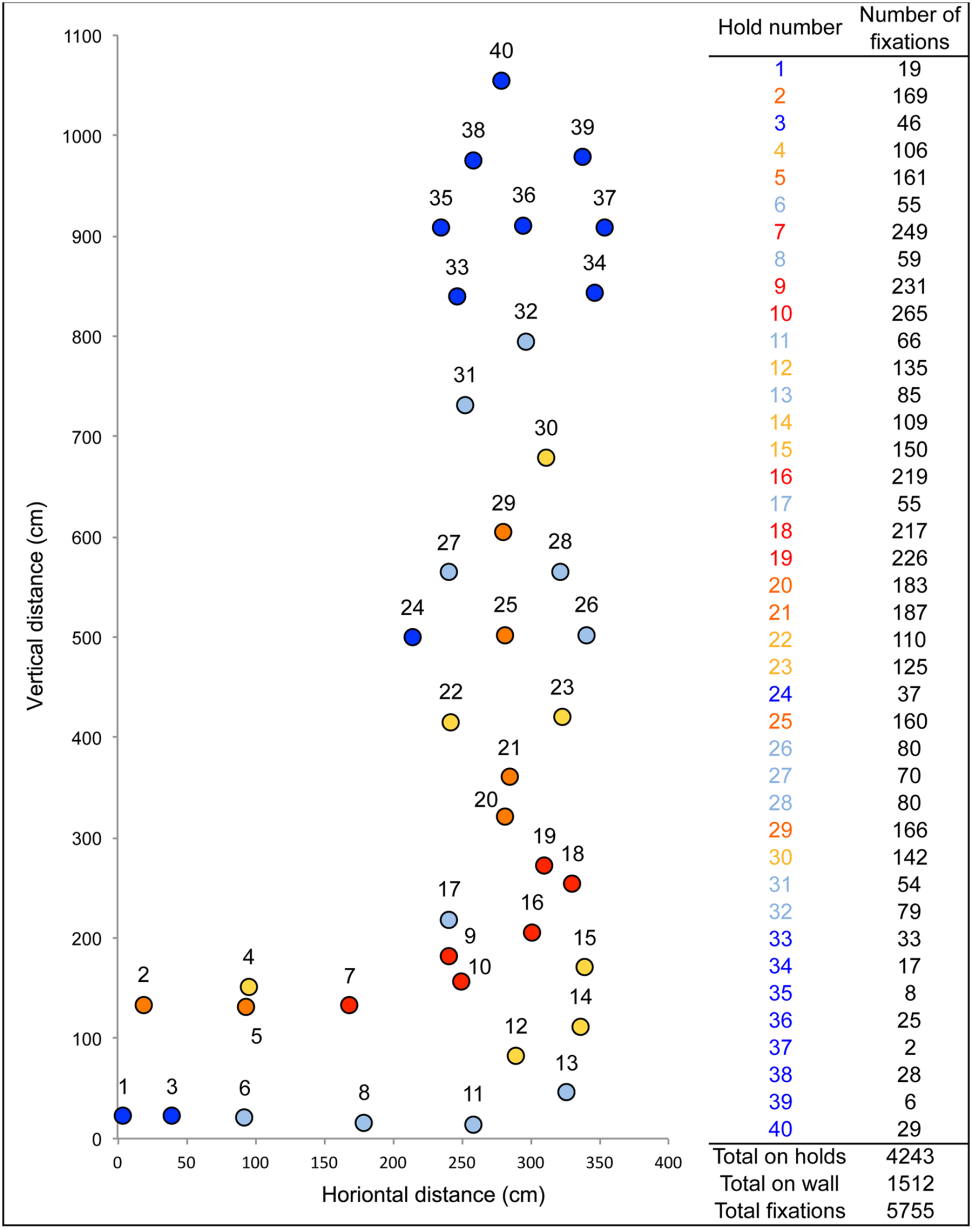
Number of fixations on each hold indicated by hold color. Red indicates lots of fixations and blue indicates few fixations. This ‘heat map’ shows that holds 7, 9, 10, 16, 18 and 19 received more attention, while holds 33–40 received little attention.

The eye tracking data quality was 84%, i.e. 84% of samples were captured. Average duration of route preview was 105.4±37.2 s, out of the maximum allowable time of 3 min. The total fixation duration was 54.6±27.2 s, which corresponded to 50.3±10.6% of the preview duration. On average there were 2.7±1.2 scan paths with an average search rate of 5.9±0.6 fixations per second. They had an average of 314±140 fixations with 233.4±106.2 fixations on holds (74±7.5% of the total number of fixations) and 80.6±41.8 fixations on the wall (26±7.5% of the total number of fixations). Next, we explored the distribution of fixations on each of the 40 holds that composed the route. Across all participants, 5755 fixations were recorded; 1512 on the wall, 4243 on holds. If the fixations had been distributed equally between all the holds, each would have had 106 fixations. Fig 3 shows the number of fixations on each hold where the hold color indicates the number of fixations on that hold. This ‘heat map’ showed that holds 7, 9, 10, 16, 18 and 19 received more attention (>200 fixations each) than the others. Hold in the highest two meters of the route received less fixations (a total of 150 fixations over all 8 holds) (Fig 3).

### Route preview profiling

Route preview profiles were determined by cluster analysis. The maximum value of CH index was for four clusters. The bootstrapping procedure showed that eight of the 13 variables differentiated the four clusters, because when these variables were excluded from the HCA they led to a modification of the number of clusters and/or of the classification of the participant in the cluster (Table 1).

**Table 1:** Modification of the number of clusters and/or the classification of the participant in the cluster when this variable was excluded from the HCA.

Variables	Best number of clusters	Number of participant switching
*Duration of the route previewing*	4	15
*Relative duration of visual fixations*	6	13
*Number of visual fixations on holds*	6	9
*Number of scans*	6	11
*Relative duration of fragmenting strategy*	4	2
*Relative duration of ascending strategy*	6	13
*Relative duration of zigzagging strategy*	4	4
*Relative duration of sequence-of-blocks*	9	9

As there were 18 participants in our sample, the maximal number of switching was 18. The number of participant switching refers to the best four clusters.

The mean and standard deviation of these eight variables are presented for the four clusters in Table 2. Ten participants were included in the first cluster, two participants in the second cluster, five participants in the third cluster and only one participant in the fourth cluster. Clusters 1 and 3 were characterized by long route previewing duration, and a high number of scan paths (>2). These two clusters also showed short relative duration of fragmenting and ascending strategies and long relative duration of zigzagging strategy and sequence-of-blocks. Clusters 1 and 3 grouped 15 of the 18 participants, and were respectively composed of four experienced and six inexperienced climbers, and three experienced and two inexperienced climbers. Clusters 2 and 4 exhibited a shorter duration of route previewing, which was associated to the smallest number of scan paths (≤2). These two clusters exhibited long relative duration of fragmenting and ascending strategies and short relative duration of zigzagging strategy. Cluster 4 had shorter duration of route preview and less visual fixations on holds compared to cluster 2, while cluster 2 spent less time previewing in sequences of blocks. Clusters 2 and 4 were exclusively composed by experienced climbers, but they only captured three participants.

**Table 2:** Mean (*M*) and standard deviation (*SD*) for the eight variables distinguishing the four clusters.

Variables	Cluster 1 n = 10			Cluster 2 n = 2			Cluster 3 n = 5			Cluster 4 n = 1		
	*M*		*SD*	*M*		*SD*	*M*		*SD*	*M*		*SD*
*Duration of the route previewing (s)*	98.2	±	10.7	59.7	±	4.5	153.4	±	9.6	28.7	±	0.0
*Relative duration of visual fixations (%)*	46.5	±	11.3	48.1	±	0.8	58.9	±	7.4	49.4	±	0.0
*Number of visual fixations on holds*	75.1	±	8.3	76.1	±	1.1	73.4	±	6.6	62.1	±	0.0
*Number of scan*	2.6	±	1.3	2.0	±	0.0	3.2	±	1.3	2.0	±	0.0
*Relative duration of fragmenting strategy (%)*	0.5	±	0.4	0.9	±	0.5	0.7	±	0.8	1.2	±	0.0
*Relative duration of ascending strategy (%)*	12.4	±	7.5	49.1	±	8.0	12.1	±	4.7	21.2	±	0.0
*Relative duration of zigzagging strategy (%)*	17.9	±	11.1	8.5	±	0.4	19.7	±	10.7	11.8	±	0.0
*Relative duration of sequence of blocks (%)*	69.2	±	12.7	41.5	±	7.1	67.5	±	8.5	65.9	±	0.0

*n* corresponds to the number of participants per cluster.

Table 3 presents the climbing duration, the climbing fluency and the relative duration of the different activity states during the climb for the four clusters.

**Table 3:** Mean (*M*) and standard deviation (*SD*) for climbing duration, climbing fluency (jerk coefficient) and the relative duration of the different activity states during the climb for the four clusters.

Variables	Cluster 1 n = 10			Cluster 2 n = 2			Cluster 3 n = 5			Cluster 4 n = 1		
	*M*		*SD*	*M*		*SD*	*M*		*SD*	*M*		*SD*
*Climbing duration (s)*	99.6	±	22.8	81.6	±	1.1	95.3	±	21.4	91.0	±	0
*Jerk coefficient*	8.0E+15	±	9.4E+14	5.7E+15	±	4.5E+14	6.9E+15	±	4.6E+14	4.0E+15	±	0
*Relative duration of immobility (%)*	33.6	±	9.5	34.4	±	7.3	43.7	±	10.6	28.1	±	0
*Relative duration of postural regulation(%)*	5.4	±	2.3	5.3	±	2.8	3.9	±	2.7	3.0	±	0
*Relative duration of hold change (%)*	37.2	±	15.3	38.3	±	3.0	29.5	±	6.2	47.3	±	0
*Relative duration of body traction (%)*	10.9	±	4.4	10.9	±	3.2	10.1	±	2.6	13.3	±	0
*Relative duration of exploration (%)*	12.9	±	7.6	11.1	±	1.8	12.7	±	3.3	8.4	±	0

*n* corresponds to the number of participants per cluster.

Pearson correlation tests showed significant correlation tests between the relative duration of exploration and the number of scans (*r* = 0.481; *p* = 0.025), between the relative duration of hold change and the number of scans (*r* = -0.416; *p* = 0.043), between the relative duration of immobility and the relative duration of zigzagging strategy (*r* = 0.457; *p* = 0.032), between the relative duration of immobility and the relative duration of sequence-of-blocks strategy (*r* = 0.426; *p* = 0.038).

## Discussion

The aim of this study was to understand the role of gaze behavior during route previewing, with a special focus on the different strategies, in order to understand whether using certain route previewing strategies (such as zigzagging and sequence-of-blocks) allowed climbers to better perceive and to realize climbing affordances. We investigated the various profiles of gaze behavior during route previewing and their relationships with the climbing ability of the participants and their climbing fluency and exploratory movements during the climb.

### What gaze behavior analysis tells us about route preview and climbing ability?

None of the climbers used the full three minutes for previewing, suggesting that they got enough information about the route to be able to climb it. In the current study we found high inter-individual variability for route preview duration, fixation duration, number of visual fixations and location of the fixations, without any obvious relationship to the level of skill of the climbers. A recent review by Dicks et al. [24] suggested that variability in gaze behavior is correlated with variability in movement coordination. In support of their suggestion, we found that various gaze behaviors (notably visual strategies) during route previewing were associated with various measures of climbing ability, fluency and movement. Route preview duration ranged from 30 s to 160 s and mainly determined the four clusters. In particular, clusters 1 and 3 showed the longest preview duration and were respectively composed of four experienced and six inexperienced climbers, and three experienced and two inexperienced climbers. Clusters 2 and 4 had the shortest preview durations and were exclusively composed by experienced climbers, but they only captured three participants.

The grade of the route corresponded to submaximal difficulty for experienced climbers and near to the maximal level of difficulty for the inexperienced climbers, but climbers from both groups often demonstrated the same previewing behavior in clusters 1 and 3. One interpretation could be that some of the experienced climbers (clusters 2 and 4) perceive the route as relatively easy and adopted corresponding visual strategies. Conversely, perhaps the inexperienced climbers and some of the experienced climbers in clusters 1 and 3 did not used visual strategies relative to route difficulty effects. Similar results indicating a route difficulty effect were reported by Pezzulo et al. [11]. In this study novice and expert climbers where asked to observe and recall the position of holds of three routes that they never climbed (easy, difficult and impossible but perceptually salient route). They showed that novice and expert climbers only differed for the difficult route; when the route was easy both groups could perform a motor simulation, and when the route was impossible neither group was able to perform a motor simulation. This interpretation seems reasonable because we did not provide the grade difficulty of the route to the climbers and they had to estimate it themselves during the preview. Moreover, route difficulty depends on the configuration of the holds (i.e. their grasp-ability) and the configuration of the limbs in transition between the holds [36]. In our study, the route difficulty was mainly due to route-finding skills, rather than grasp-ability of holds, because the climbers had to cope with a tricky climbing path (i.e. a traverse and a succession of two hourglasses). For instance, Fig 3 shows clearly that the transition between the horizontal and vertical sections of the route received the highest number of fixations, possibly because the route and the use of holds are not obvious. This suggests that route finding involves perceiving the action possibilities (affordances) and acquiring this skill may be facilitated by route previewing. Although climbers move along the climbing wall and simulate actions during the pre-visual inspection of the route, the analysis of route previewing should be linked to the analysis of the climb to better understand its impact on affordances perception and realization.

### What gaze behavior analysis tells us about visual strategies and route preview profiling?

The cluster analysis highlighted three main findings regarding the visual strategies and route preview profiling: (i) The first profile exhibited longer relative duration of zigzagging strategy, higher number of scans and longer relative duration of fixation and longer duration of route preview (mainly represented by cluster 3 in priority, then cluster 1). (ii) The second profile showed longer relative duration of an ascending strategy, a smaller number of scans and shorter duration of route preview (mainly represented by clusters 2 and 4). (iii) Detailed visual analysis corresponded to sequence-of-blocks, which exhibited the longest visual strategy duration in all the clusters of climbers.

**Fig 4:**
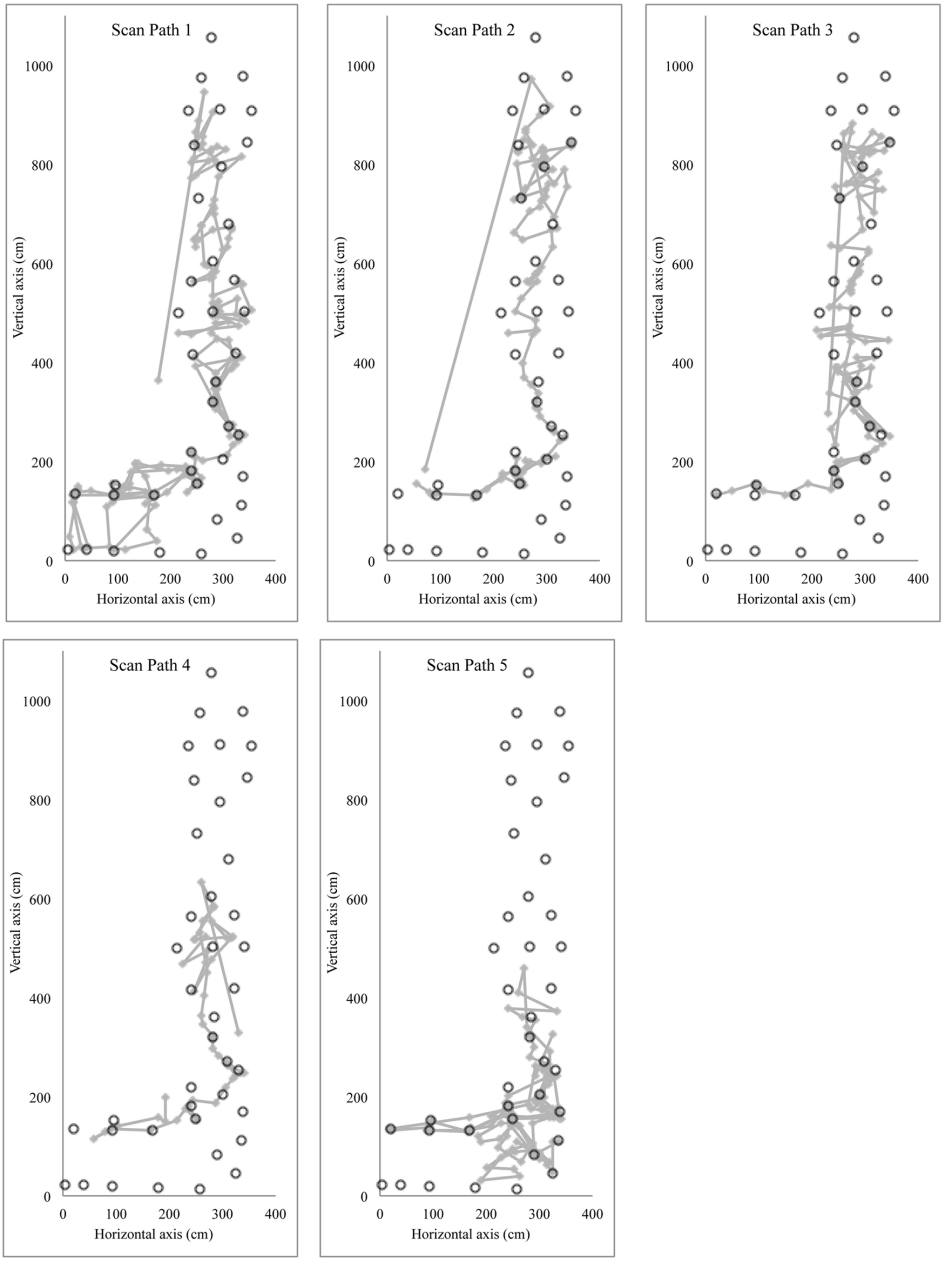
Pictogram of the visual fixations for participant 6, doing five scans path. Rings represent the 40 holds of the route.

The first profile includes clusters 1 and 3, which mainly differed from clusters 2 and 4 by the duration for the route preview. They generally had a higher number of scan paths, which were used to *find the route*, then the longer relative duration of fixation, and finally a longer relative duration of a zigzagging strategy. Route finding skill requires “*a series of well-formed movements which are linked together into actual ‘sentences’ and serve to structure the climber’s motor behavior*” (p. 371) [14]. In other words it entails rapidly and correctly find the climbing path [13,14,37] and the way to grasp holds [4,6]. Poor route finding skill might lead to more exploration both at motor [4–6] and visual levels [26], which may require several scan paths while previewing the route. Fig 4 shows an example for participant 6 in cluster 3 who required five scan paths to find the route during a preview of 155 s with 600 fixations. The fifth scan path was fully dedicated to the transition between the horizontal and vertical sections.

The longer duration of route preview of the climbers in clusters 1 and 3 might also come from *longer relative duration of fixation*, which implies that the climbers were spending more time interpreting because they might have difficulty in picking up functional information [38], i.e. perceiving the specifying information for action [39]. Boschker et al. [5] highlighted that inexperienced climbers exclusively perceived structural features of holds when looking at a climbing wall (e.g., location, shape, and orientation) whereas experienced climbers mainly focused on the functional features, such as the grasping, reaching, and standing opportunities of individual holds as well as the chains of climbing moves using multiple holds. Therefore, picking up functional information might also explain the use of *zigzagging strategy*, which involves a series of upward and downward visual fixations. In fact, a zigzagging strategy might occur when climbers were simulating the passing from one hold to the next, i.e. climbers perceived chaining actions of the hands and feet, as well as the chaining hand actions. Boschker and Bakker [6] reported that inexperienced climbers used a ‘hold-to-hold’ approach such as dual grasping rather than a more complex coordination such as arm crossing. These findings suggested that climbers might used a zigzagging strategy while previewing when the holds did not afford clear coordination between actions. Indeed, as discussed in the next section, the relative duration of zigzagging strategy during route previewing was positively correlated to the relative duration of immobility during climbing, supporting that this strategy might revealed deep visual analysis [17].

**Fig 5:**
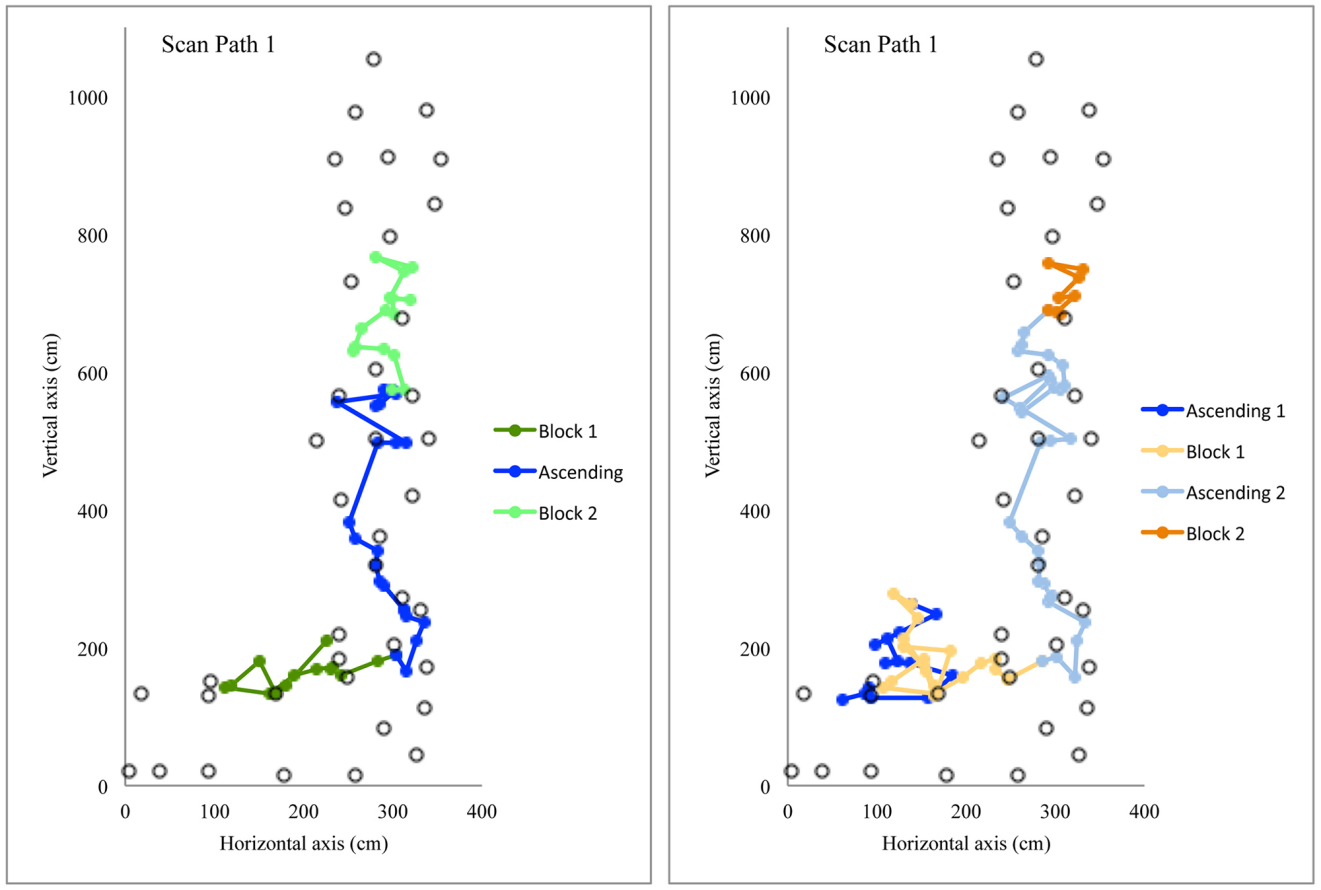
Pictogram of the visual strategies for participants 3 and 5, using mainly *ascending* and *sequence-of-blocks* strategy during their first scan path. The grey circles represent the 40 holds of the route.

The second profile includes the climbers of clusters 2 and 4 who exhibited a shorter duration of route preview, longer relative duration of an ascending strategy and a smaller number of scan paths. According to Grushko and Leonov [17], an ascending strategy corresponds to a general visual inspection to get an overview of the route or a section of the route. Fig 5 shows the first scan of two participants in cluster 2 who alternated between an ascending strategy with sequence-of-blocks during a relatively short first scan path; participant 3 (50 fixations over a duration of 7.9 s) and participant 5 (72 fixations over a duration of 11.3 s). According to the ecological approach of visual perception [3], it could be postulated that climbers who chain their visual fixations through the route clearly perceive possibilities of action (affordances) offered by the holds. Indeed, the climbers within clusters 2 and 4 used only two scan paths, suggesting that they did not need additional information for action. As discussed previously, experienced climbers in clusters 2 and 4 are sensitive to constraints influencing relative difficulty and adopt a general visual inspection because they perceived the route properties as relatively easy (i.e., well within their capabilities).

**Fig 6:**
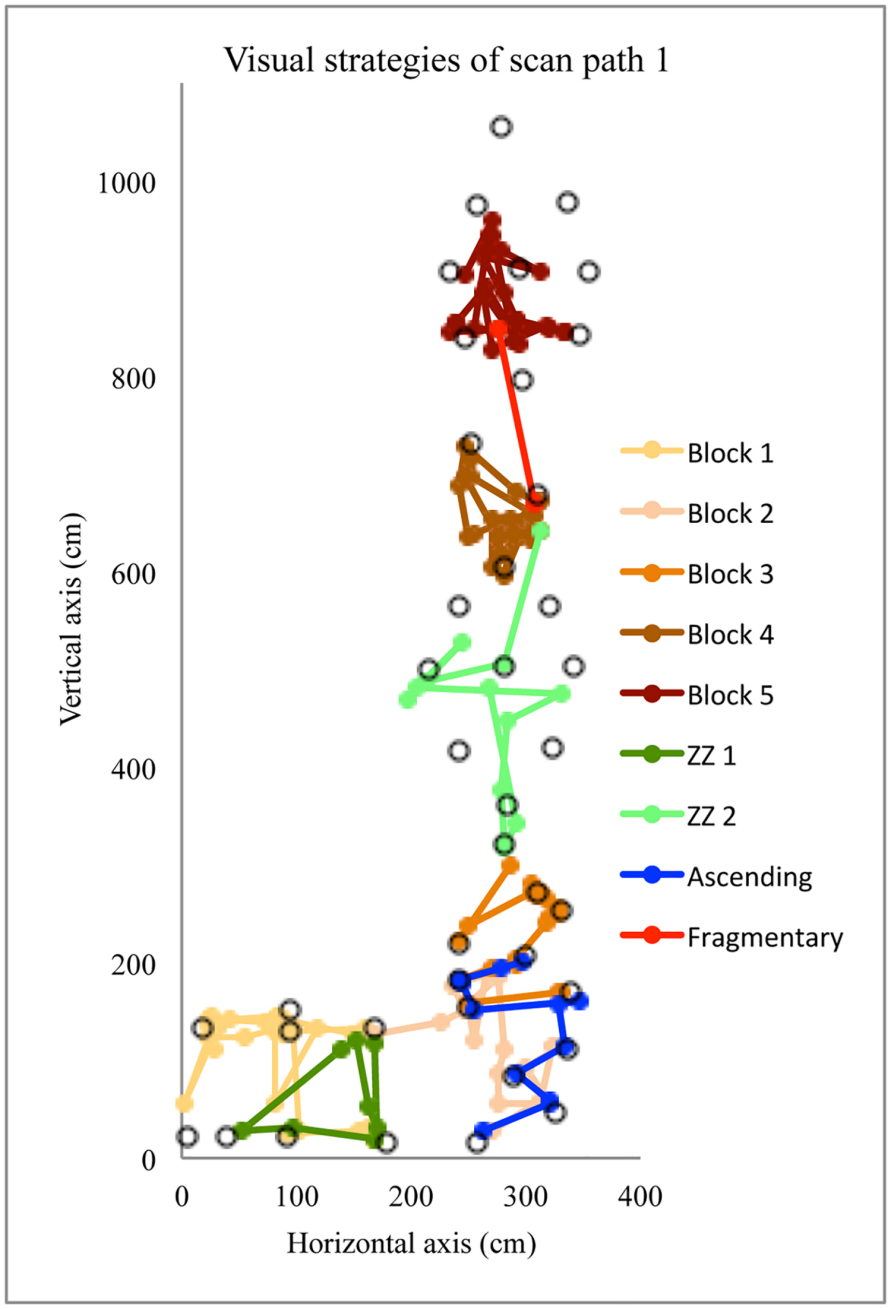
Pictogram of the visual strategies for participant 14, using mainly *sequence-of-blocks* strategy during his first scan path. The grey circles represent the 40 holds of the route.

According to Grushko et al. [17], the sequence-of-blocks strategy reflects deep visual analysis and mainly occurs when climbers perceive a crux region (i.e., difficult part of the route). Previewing a crux region might reflect the difficulty to grasp or to use a hold, or to chain two holds together, but probably not longer chains of movements. Thus, the sequence-of-blocks strategy could reflect the difficulty of climbers to pick up information for perceiving hold functionality, such as how to grasp or support progression [5]. In a study where the participants had to categorize hold functionality, Bläsing et al. [40] showed that the categorization of holds was influenced by their potential for interaction. Thus, only certain features of holds (e.g., hold orientation, pocket size—open to insert one or more fingers, one or more phalanges) were found to determine their potential use and are, therefore, relevant for functional categorization (e.g. grasping types), whereas other features play a minor role (e.g. shape and color) [40]. They found that inexperienced climbers were less able than experienced climbers to decide consistently which specific type of grasp was required for given holds [40]. Conversely, experienced climbers could decide more rapidly and with more congruence about the grasp-ability of holds (e.g. crimp grasping vs. sideways pull) [40]. Therefore, the sequence-of-blocks strategy might be used when the climbers have difficulty in categorizing the hold functionality and to perceive opportunities for action. Climbers of clusters 1 and 3 probably used sequence-of-blocks strategy to determine the grasp-ability of holds and the climbing path. For instance, Fig 6 shows an example of participant 14 in cluster 1, who mainly used a sequence-of-blocks strategy during the first scan of his preview (170 fixations over a duration of 29.1 s). It is evident that the hourglass design, which offers multiple paths, requires ‘route finding’ skill and leads to a sequence-of-blocks strategy (sequence-of-blocks 5 in Fig 6). We can also observe sequence-of-blocks (sequences of blocks 2 and 3 in Fig 6) probably because the grasp-ability of the holds and the chain between holds to transition between horizontal and vertical sections was not obvious to this climber. This is similar to the route used by Pezzulo et al. [11], which he reported was difficult to climb because the holds were not easily graspable due to their shape and orientation, with only expert climbers being attuned to their grasp-ability [11].

### What route preview tells us about climbing fluency and exploratory movements?

Climbers in clusters 2 and 4 with the shorter duration of route preview tended to climb more fluently (i.e., lower jerk coefficient) than climbers in clusters 1 and 3, notably because they exhibited shorter climb duration, lower relative duration of immobility, lower exploration with limbs, and longer relative duration spent transiting from one hold to anther (Table 3). Our results support previous findings showing that route preview seems to influence ‘form performance’ [12]; climbers made fewer and shorter stops during their ascent following certain preview strategies, perhaps allowing them to chain climbing movements and climb more fluently [12].

However, jerk coefficient was highly variable within the clusters so correlation tests between route preview variables and climbing variables were performed on all the climbers together. The Pearson correlation tests emphasized that a high number of scan paths was associated with a high relative duration of exploration and a low relative duration of hold change during the ascent. Additionally, a high relative duration of sequence-of-blocks and zigzagging strategies was related to a high relative duration of immobility during the ascent. As discussed previously, route previewing allows a climber to find the route path, but also to pick up functional information about reachable, graspable and usable holds, in order to chain movements together. A high number of scan paths, a long sequence-of-blocks and zigzagging strategies during the preview have been shown to reflect deep analysis probably because the route did not afford opportunities for action for the climbers, and consequently use many exploratory movements and have many immobile periods. Gibson [2] suggested that “*using even a simple tool* (like a climbing hold) *is at a minimum a two-step event–an action that serves as a means to a further step of reaching something desirable*” p.25). Further, she described the role of ‘ambulatory exploration’ as a primary function of perception to guide locomotion in infants [2]. Climbing is a form of quadrupedal locomotion on a vertical plan that an adult experiences through exploratory activities in a similar way that an infant discovers when he/she learns to walk. Thus, we move to reach and we reach to grasp, and it could be added, we grasp to use; it suggests that experienced climbers perceived ‘*clustered’ affordances* or even *‘nested’ affordances* (such as reaching, grasping, standing and climbing possibilities) [5,10]. In a memory recall task, Boschker et al. [5] proposed that climbers had picked up ‘clustered’ information if they recalled more than nine items after the 5 s viewing period. The authors indicated that the expert climbers recalled a large amount of information that reflected relevant features for climbing, supporting their ability to perceive moves in which more than one hold is involved and at which several reaching, grasping or standing possibilities are perceived as one (clustered) climbing affordance [5]. However, ‘clustered’ implies a purely spatial conceptualization of information, whereas a term like ‘*nested’* addresses temporal issues (such as prospection, i.e., forward-looking [41]). In fact, in the memory recall tasks by Boschker et al. [5], the climbers were not asked to climb the route afterwards, the task was purely memory recall, and perhaps in this task context, the idea of a clustered affordance was a useful term. The problem of considering how time influences the nature of affordance perception is one that our study attempted to address by investigating how holds were chained in time during different previewing strategies. Indeed, the climbers usually take an ordered progression through the route during route previewing (e.g. bottom to top), and are not only concerned with spatial information (e.g., memorizing chunks of the climbing wall). Surprisingly, in the memory recall tasks by Boschker et al. [5], the climbers started their recall from the top of the route, which is not common practice when previewing before a climb.

We hypothesize that the role of route preview is to optimize the picking up of information for the perception of *nested affordances*, to maximize the number of performatory actions (i.e., actions used as support to move of the center of mass) and minimize the number of exploratory actions (i.e., actions which did not support the displacement of the center of mass). Similarly, Hörst [42] made several recommendations to optimize route previewing: (i) route previewing should be performed from different locations to get different points of view on hold grasp-ability and the route path; (ii) route previewing should help to chain movements together in a ‘sequence’ (also advised by [43]); (iii) rest points should be perceived to enable to support strategic behavior such as rest and back up (like climbing down) but also to revise the initial ‘sequence’ of movements; (iv) crux points should be perceived in order to explore possibilities to climb them.

In conclusion, the ecological approach of visual perception emphasizes that perceiving is already acting and we perceive through action. Even though route previewing (and mental imagery) do not replace climbing itself, it enables a climber to attune to the invariants that afford action, i.e. a combination of features, such as hold orientation, shape, texture, and size afford certain grasps. In other words, route previewing might help a climber to perceive compound invariants (a unique combination of invariants) on the climbing wall that invite action possibilities (affordances). At a higher level, a combination of holds with certain features provide compound invariants that afford certain body positions when a climber transitions between holds, suggesting that the perception of *‘nested’ affordances* (such as reaching, grasping, standing, and climbing possibilities) might indicate previewing skill. Constraints that induce switching from perceiving opportunities for passing between holds toward their grasp-ability may be particularly pertinent for learning design. For instance, anxiety during climbing narrows attention towards the climber’s body and increases the number of hold related exploratory actions [7] and fixations [26]. Perhaps specific preview strategies can help prevent these negative effects of anxiety.

## Supporting information

S1 File Coordinates of the 40 holds. (XLS)

S2 File Coordinates of the visual fixations, visual fixations duration, distance between visual fixation and nearest climbing hold for each individual. (XLSX)
